# 1225. Optimization of Antimicrobial Therapy for Extended-Spectrum β-Lactamase-Producing *Enterobacterales* (ESBL-E) Bloodstream Infections Through Laboratory-Based Interventions

**DOI:** 10.1093/ofid/ofad500.1065

**Published:** 2023-11-27

**Authors:** Mona Yazdi, Rebecca Wang, Isabella W Martin

**Affiliations:** Dartmouth Health, MISSION VIEJO, California; Dartmouth-Hitchcock Medical Center, Lebanon, New Hampshire; Dartmouth-Hitchcock Medical Center, Lebanon, New Hampshire

## Abstract

**Background:**

In 2020, the Infectious Diseases Society of America (IDSA) published clinical practice guidance recommending up-front treatment with an IV carbapenem for severe infections with extended-spectrum β-lactamase (ESBL)-producing Enterobacterales (EB).^1^ This single-center, observational study sought to optimize adherence to IDSA guidance through two laboratory-based interventions.

**Methods:**

On 12/29/2021, the laboratory initiated the first intervention: suppression of piperacillin-tazobactam results for EB isolates with ceftriaxone resistance, a surrogate for ESBL production.^2^ On 5/16/2022, the laboratory adopted the second intervention: implementation of a new rapid sepsis diagnostic assay (from BCID to BCID2, BioMerieux). BCID2 detection of *bla*_CTX-M_ was accompanied by a report footnote recommending up-front carbapenem therapy. To determine the impact of these interventions, patients with blood cultures positive for ceftriaxone-resistant EB between 1/1/2019 and 11/7/2022 were identified. Chart review was performed to determine demographic data, antimicrobial use, and clinical outcomes. The time period between the first and second interventions was excluded. The primary endpoint was use of an IV carbapenem within 24 hours of available phenotypic antimicrobial susceptibility test results.

**Results:**

During this time period, a total of 44 unique ceftriaxone-resistant EB were isolated in blood culture. Of these, 34/44 (77.3%) were *Escherichia coli*, 6/44 (13.6%) were *Klebsiella pneumoniae*, and 4/44 (9.1%) were *Klebsiella oxytoca*. The primary endpoint was observed in 19/33 (57.6%) patients prior to the interventions and 11/11 (100%) patients after.

**Conclusion:**

Through serial laboratory-based interventions, we successfully improved institutional adherence to IDSA guidance for treatment of ESBL-EB bloodstream infections. The findings of our study may inform collaborative microbiology-stewardship efforts at other facilities.

**Disclosures:**

**All Authors**: No reported disclosures

